# *Artemisia indica* Willd. Extract Regulate NLRP3 Inflammasome and ENaC Trafficking in Angiotensin II-Stimulated Renal Tubular Cells

**DOI:** 10.3390/plants15091405

**Published:** 2026-05-04

**Authors:** Chiao-Yun Tseng, Hui-Hsuan Lin, Yu-Hsuan Liang, Chia-Wen Tsai, Yueching Wong, Jing-Hsien Chen

**Affiliations:** 1 Department of Nutrition, Chung Shan Medical University, Taichung City 40201, Taiwan; 1146002@live.csmu.edu.tw (C.-Y.T.); 1346003@live.csmu.edu.tw (Y.-H.L.); wyc@csmu.edu.tw (Y.W.); 2 Department of Medical Laboratory and Biotechnology, Chung Shan Medical University, Taichung City 40201, Taiwan; linhh@csmu.edu.tw; 3 Department of Nutrition, China Medical University, Taichung City 406040, Taiwan; cwtsai@mail.cmu.edu.tw; 4 Department of Medical Research, Chung Shan Medical University Hospital, Taichung City 40201, Taiwan

**Keywords:** *Artemisia indica* Willd., isochlorogenic acid C, epithelial sodium channel, NLRP3 inflammasome, angiotensin II

## Abstract

*Artemisia indica* Willd. is widely used in traditional medicine and dietary practices. Phytochemical analysis of *Artemisia indica* Willd. aqueous extract (AAE) by HPLC–ESI–MS/MS identified isochlorogenic acid C (ICAC) as a major constituent. Angiotensin II (Ang II) disrupts renal tubular epithelial cell homeostasis and contributes to renal injury. In this study, we evaluated the protective effects of AAE and ICAC in Ang II-stimulated NRK52E cells. Both AAE and ICAC significantly reduced reactive oxygen species (ROS) production, mitochondrial dysfunction, and proinflammatory cytokine release. Mechanistic analyses showed that AAE inhibited Ang II type 1 receptor (AT1R)-mediated NF-κB activation and suppressed NLRP3 inflammasome signaling, thereby alleviating inflammatory responses and pyroptosis. In addition, AAE and ICAC restored sodium homeostasis by reactivating neural precursor cell expressed developmentally downregulated gene 4-like (Nedd4-2), promoting epithelial sodium channel (ENaC) ubiquitination and reducing its apical membrane accumulation. Molecular docking suggested that ICAC interacts with the extracellular domain of α-ENaC, supporting its regulatory role. Overall, AAE and ICAC protect renal tubular epithelial cells against Ang II-induced injury by reducing oxidative stress, inflammation, and dysregulated sodium transport, highlighting their potential as plant-derived therapeutic agents for hypertension-associated renal dysfunction.

## 1. Introduction

Although chronic kidney disease (CKD) has multiple etiologies, hypertension is particularly prevalent in Asian populations [1]. Elevated blood pressure leads to progressive renal injury, establishing a vicious cycle in which kidney dysfunction further sustains hypertension [2]. The pathophysiological mechanism underlying this process is the excessive activation of the renin–angiotensin system (RAS), characterized by increased production of angiotensin II (Ang II) [3]. Ang II is generated through a cascade of enzymatic reactions involving renin and angiotensin-converting enzyme (ACE), and it exerts its effects by binding to angiotensin type 1 receptors (AT1R) [4]. In addition to its hemodynamic effects, Ang II acts as a cytokine-like mediator with potent proinflammatory properties. At the tubular level, it promotes sodium and water retention, cellular hypertrophy, and oxidative stress, thereby amplifying renal injury [5]. Ang II promotes sodium reabsorption in the proximal tubule through activation of the basolateral Na^+^/K^+^-ATPase, thereby contributing to elevated blood pressure [6]. This interaction activates protein kinase C (PKC), initiating downstream cascades linked to inflammation and oxidative stress [7]. Ang II promotes NF-κB activation through both PKC-dependent and ROS-mediated mechanisms, leading to the transcription of proinflammatory genes, including interleukin-1 beta (IL-1β), and interleukin 6 (IL-6) [8]. Persistent activation of PKC and NF-κB drives oxidative stress, vascular injury, and organ remodeling, thereby promoting the progression of hypertension and endothelial dysfunction [9]. At the renal level, the epithelial sodium channel (ENaC), a heterotrimeric protein expressed in epithelial tissues such as the kidney, lung, and colon, plays an essential role in salt and water homeostasis [10]. ENaC assembly and surface expression are tightly regulated by neural precursor cell expressed developmentally downregulated gene 4-like (Nedd4-2). Phosphorylation of Nedd4-2 inhibits its ubiquitin ligase activity, thereby stabilizing ENaC at the cell membrane and enhancing sodium reabsorption [11]. Consistent with this, loss of Nedd4-2 in mice results in salt-sensitive hypertension and increased renal ENaC expression [4].

*Artemisia* is a diverse and economically important genus within the *Asteraceae* family that has long been valued in folk medicine and as a dietary plant across various cultures [12,13]. Among these, *Artemisia indica* has been widely reported as both a culinary herb and a food plant in regions such as India, Nepal, and Japan. *Artemisia* species have a long history of dietary and medicinal use and are recognized for their antioxidant properties [14]. Ethnomedicinally, *Artemisia indica* Willd. has been traditionally employed to alleviate chronic fever, dyspepsia, and hepatobiliary disorders [13,15], while its essential oil has also been proposed as a natural preservative to enhance food safety and shelf life [16,17]. Ethnopharmacological surveys from regions such as Pakistan and India have documented the traditional use of *Artemisia* species for the management of hypertension among local populations [18]. In addition, previous studies have shown that *Artemisia* species, such as *Artemisia vulgaris* L., *Artemisia deserti* and *Artemisia herba alba*, exert significant antihypertensive and vasorelaxant effects in animal models. However, the underlying molecular mechanisms remain incompletely understood [19,20,21]. Furthermore, *Artemisia indica* Willd. is widely distributed in tropical and subtropical regions and is commonly consumed as a traditional vegetable, highlighting its potential as a functional food [22]. Owing to their rich phytochemical diversity, particularly in terpenoids, flavonoids, and phenolic acids, *Artemisia* species have garnered considerable scientific attention. However, despite their widespread ethnomedicinal use, certain species remain comparatively under-investigated. With the growing interest in herbal medicines as complementary and alternative therapies, *Artemisia* species have garnered increasing attention due to their safety, affordability, and potential effectiveness compared to synthetic drugs [23,24]. Based on these considerations, this study aimed to investigate whether AAE and its major constituent exert protective effects against Ang II-induced renal injury and to elucidate the underlying molecular mechanisms.

## 2. Results

### 2.1. Cytoprotective Effects of AAE Against Ang II-Induced Cell Damage

**Figure 1:**
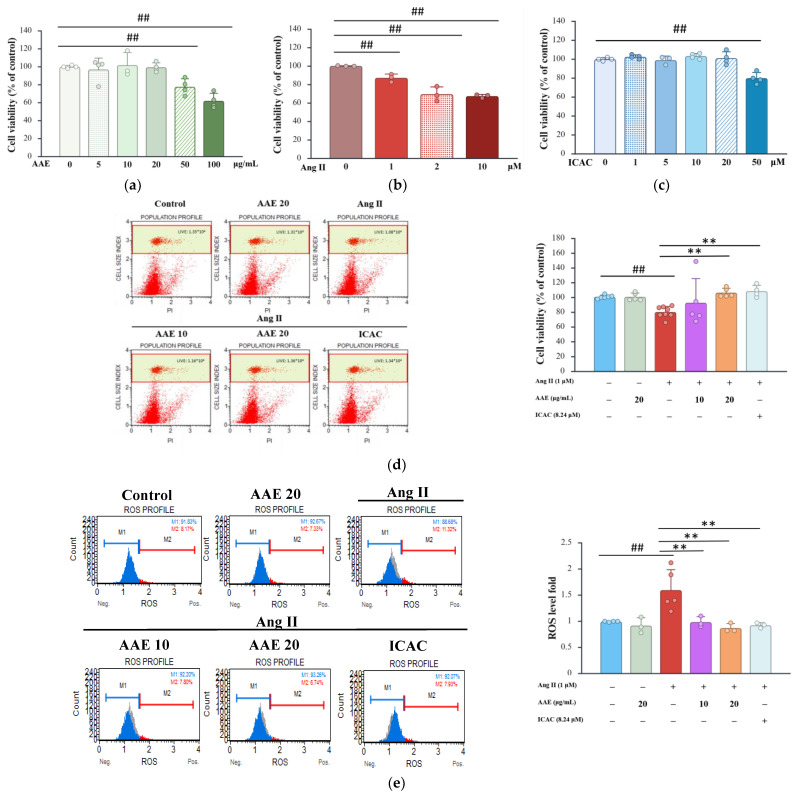
Cytoprotective effects of AAE against Ang II-induced cell damage. (**a**) Quantification of NRK52E cells treated with various concentrations of AAE (0, 5, 10, 20, 50, and 100 μg/mL) for 24 h. (**b**) Quantification of NRK52E cells involved various concentrations of Ang II (0, 1, 2, and 10 μΜ) for 24 h. (**c**) Quantification of NRK52E cells involved various concentrations of ICAC (0, 1, 5, 10, 20 and 50 μΜ) for 24 h. (**d**) NRK52E cells pretreated with AAE (10 and 20 μg/mL) and ICAC (8.24 µM) for 1 h, then a combination of Ang II (1 μΜ). Cell viability was assessed using propidium iodide (PI) staining and analyzed with a Muse™ Cell Analyzer. (**e**) The ROS production was assessed using an oxidative stress detection kit and analyzed by flow cytometry. The quantitative data were presented as the mean ± SD, and each dot represents an independent experimental replicate. ## *p* < 0.01 vs. control; ** *p* < 0.01 vs. Ang II, analyzed by one-way ANOVA with Tukey’s post hoc test.

The results indicated that AAE exhibited no cytotoxic effects at concentrations of 10 and 20 μg/mL (Figure 1a). However, exposure to Ang II at 1 μM significantly reduced cell viability (Figure 1b). ICAC exhibited no cytotoxic effects on NRK52E cells at concentrations up to 20 μM (Figure 1c). To investigate the potential cytoprotective role of AAE, NRK52E cells were pretreated with AAE and ICAC before being induced with Ang II. Flow cytometry analysis confirmed that AAE effectively restored cell viability following Ang II treatment (Figure 1d). Furthermore, Ang II was found to increase ROS production significantly, but AAE treatment significantly mitigated this effect (Figure 1e). These findings suggest that AAE exerts protective effects against Ang II-induced oxidative stress and cell damage.

### 2.2. Protective Effects of AAE and ICAC Against Ang II-Induced Mitochondrial Dysfunction and Inflammation

**Figure 2:**
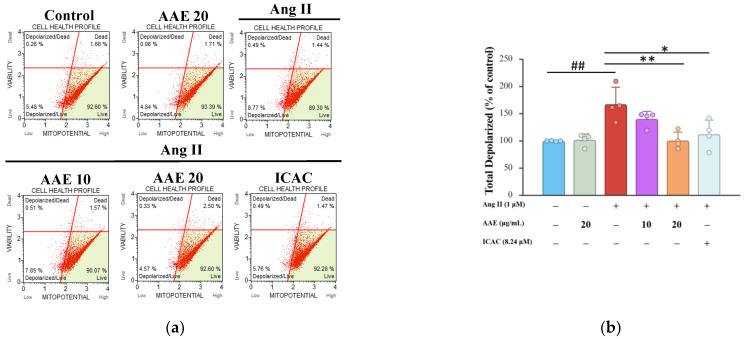

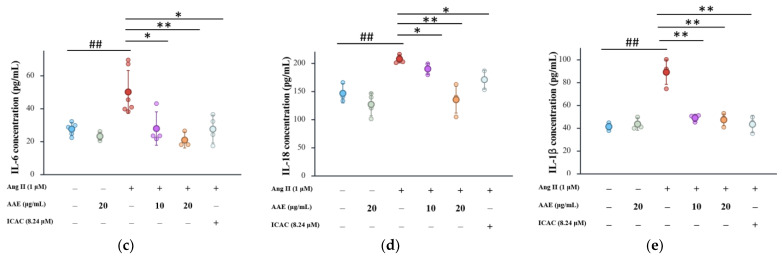
Protective effects of AAE and ICAC against Ang II-induced mitochondrial dysfunction and inflammation. (**a**) Mitochondrial membrane potential was evaluated using the JC-1 assay and analyzed with a Muse™ Cell Analyzer. The *x*-axis represents mitochondrial membrane potential (MitoPotential dye), while the *y*-axis represents cell viability (7-AAD staining). (**b**) Quantitative analysis of the ratio of total depolarization intensity. (**c**) The inflammatory cytokines, including IL-6, IL-18 (**d**), and IL-1β (**e**) were detected by ELISA. The quantitative data were shown as mean ± SD, and each dot represents an independent experimental replicate (*n* ≥ 3). ## *p* < 0.01 vs. control; * *p* < 0.05 and ** *p* < 0.01 vs. Ang II, analyzed by one-way ANOVA with Tukey’s post hoc test.

Ang II induces ROS production, contributing to pathological conditions, including organ damage in hypertension. In the bivariate plots, the *x*-axis represents mitochondrial membrane potential (MitoPotential dye), while the *y*-axis represents cell viability (7-AAD staining). Our results indicated that Ang II exposure increased mitochondrial depolarization, a key marker of mitochondrial dysfunction (Figure 2a). However, treatment with AAE and ICAC significantly reduced mitochondrial depolarization, with high doses of AAE providing the most significant protective effect (Figure 2b). Ang II-induced mitochondrial depolarization contributed to oxidative stress, which in turn activated inflammatory signaling pathways, leading to the production of proinflammatory cytokines, including IL-6 (Figure 2c), IL-18 (Figure 2d), and IL-1β (Figure 2e). Modulation of these cytokines may contribute to the management of hypertension-associated inflammation. Ang II significantly increased cytokine release, whereas AAE and ICAC attenuated this inflammatory response.

### 2.3. AAE Inhibits Ang II-Induced PKC/NF-κB Activation and NLRP3 Inflammasome Signaling

**Figure 3:**
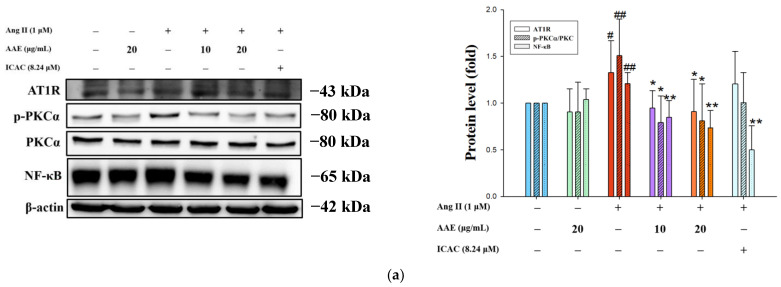

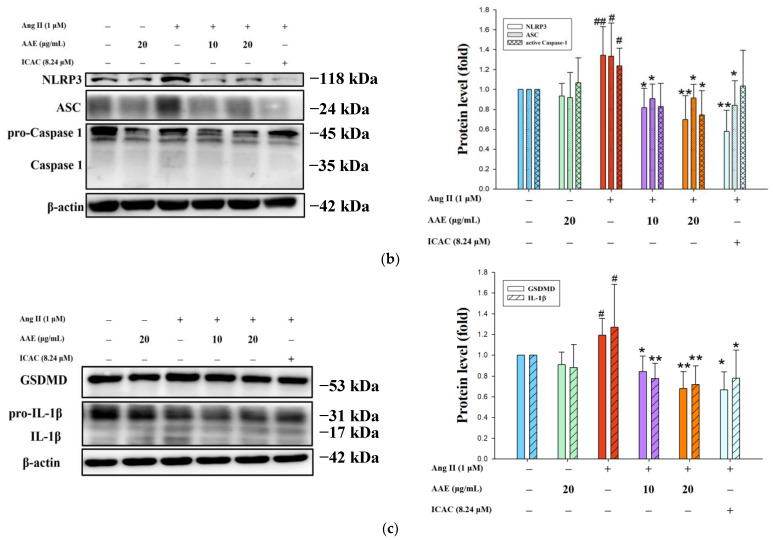
AAE inhibits Ang II-induced PKC/NF-κB activation and NLRP3 inflammasome signaling. Protein expression levels of AT1R, p-PKCα, PKCα, and NF-κB (**a**); NLRP3, ASC, and caspase-1 (**b**); and GSDMD and IL-1β (**c**) were determined using Western blot analysis. β-actin served as an internal control. The quantitative data were shown as mean ± SD (*n* ≥ 3), derived from at least three independent biological replicates. # *p* < 0.05 and ## *p* < 0.01 vs. control; * *p* < 0.05 and ** *p* < 0.01 vs. Ang II, analyzed by one-way ANOVA with Tukey’s post hoc test.

Ang II activated PKC through its interaction with the AT1R, thereby enhancing NF-κB activation, which subsequently regulated the transcription of proinflammatory cytokines. As shown in Figure 3a, AAE effectively inhibited the expression of AT1R, PKC, and NF-κB. Our study observed increased expression of inflammasome (Figure 3b); however, these effects were reversed by AAE and ICAC treatment (Figure 3c). Overall, our results suggest that AAE inhibits PKC activation and suppresses NF-κB signaling, thereby downregulating NLRP3 inflammasome activation and mitigating Ang II-induced inflammatory responses through attenuation of pyroptosis.

### 2.4. Ang II Modulation of Na-K-ATPase and SGK1 in NRK52E Cells

**Figure 4:**
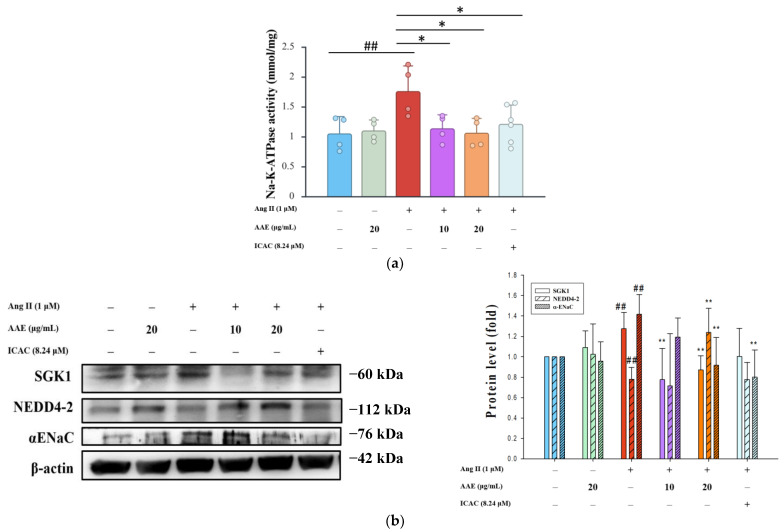
Ang II modulation of Na-K-ATPase and SGK1 in NRK52E cells. (**a**) Quantification of Na-K-ATPase activity. The protein levels of SGK1, NEDD4-2, and α-ENaC (**b**) were analyzed by Western blotting. β-actin served as an internal control. The quantitative data were shown as mean ± SD, and each dot represents an independent experimental replicate (*n* ≥ 3). ## *p* < 0.01 vs. control; * *p* < 0.05 and ** *p* < 0.01 vs. Ang II, analyzed by one-way ANOVA with Tukey’s post hoc test.

However, this stimulation was significantly reduced by AAE and ICAC, suggesting their involvement in modulating ion channel activity (Figure 4a). In addition to its role in Na-K-ATPase regulation, serum and glucocorticoid-regulated kinase 1 (SGK1) expression is also upregulated in response to Ang II and is influenced by multiple signaling pathways, including PKC [25]. Interestingly, AAE treatment led to a decrease in SGK1 expression while upregulating Nedd4-2, a well-known regulator of α-ENaC. Furthermore, both AAE and ICAC treatments suppressed α-ENaC expression, likely as a protective mechanism to prevent excessive sodium influx (Figure 4b).

### 2.5. Regulation of AAE Treatment on α-ENaC Expression and Ubiquitination in NRK52E Cells

**Figure 5:**
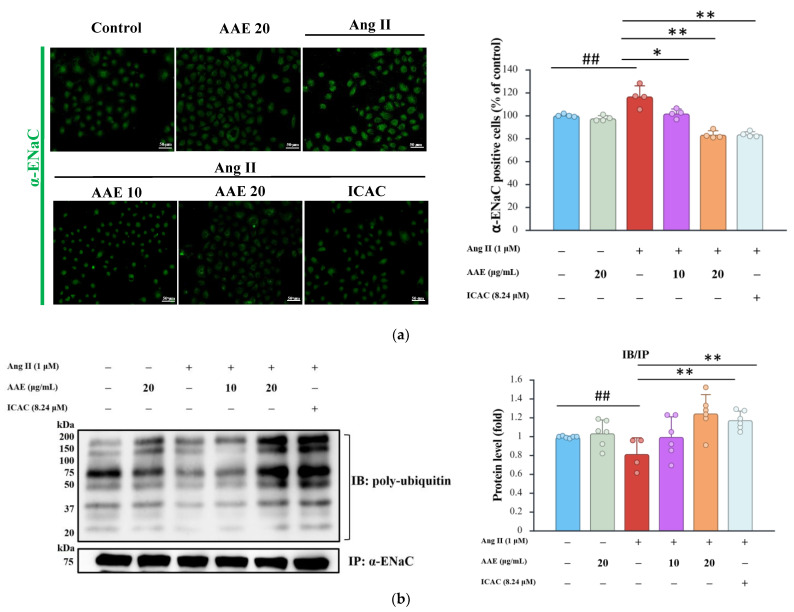
Regulation of AAE treatment on α-ENaC expression and ubiquitination in NRK52E cells. (**a**) Image of immunocytochemistry staining with α-ENaC and quantitation. Scale bar, 50 μm. (**b**) The protein levels of ubiquitin and α-ENaC were analyzed by IP. The cell extracts were IP with α-ENaC. The quantitative data were shown as mean ± SD, and each dot represents an independent experimental replicate (*n* ≥ 3). ## *p* < 0.01 vs. control; * *p* < 0.05 and ** *p* < 0.01 vs. Ang II, analyzed by one-way ANOVA with Tukey’s post hoc test.

Our findings demonstrate that treatment with AAE and ICAC markedly reduced α-ENaC fluorescence intensity, as assessed by immunocytochemistry (Figure 5a), indicating diminished α-ENaC abundance at the cellular level. To further delineate the regulatory mechanism underlying this effect, we examined α-ENaC ubiquitination in NRK52E cells. Notably, high-dose AAE and ICAC treatments significantly enhanced α-ENaC ubiquitination (Figure 5b), suggesting activation of ubiquitin-dependent proteasomal degradation pathways. These findings suggest that AAE and ICAC promote α-ENaC degradation via the ubiquitin–proteasome pathway, thereby reducing its membrane abundance and limiting sodium influx. Under Ang II stimulation, SGK1-mediated inhibition of Nedd4-2 suppresses α-ENaC ubiquitination, thereby sustaining α-ENaC surface expression and promoting persistent sodium reabsorption. Consistent with these findings, Figure 4b shows that Ang II markedly activated SGK1, thereby suppressing Nedd4-2 activity. Importantly, this dysregulation signaling axis was effectively reversed by AAE and its active constituent ICAC, restoring Nedd4-2-dependent α-ENaC. These findings indicated a previously underappreciated role of AAE and ICAC in re-establishing dynamic α-ENaC homeostasis through coordinated modulation of SGK1–Nedd4-2 signaling, rather than merely suppressing α-ENaC expression, thereby providing a mechanistic basis for their sodium-lowering and renoprotective effects under Ang II-driven stress conditions.

### 2.6. Molecular Docking Analysis of ICAC with α-ENaC

**Figure 6:**
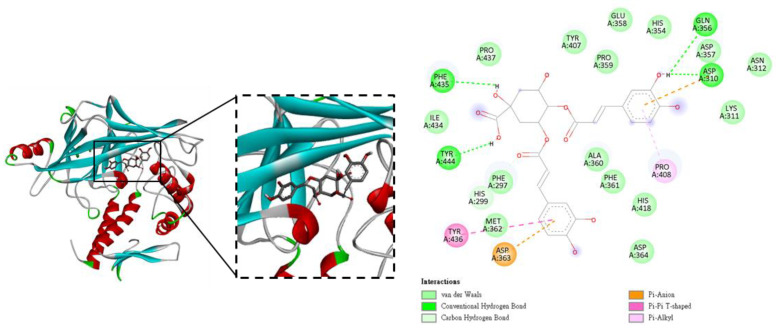
Molecular docking analysis of ICAC with α-ENaC. Two-dimensional molecular docking images illustrate the predicted binding mode and structural interactions of ICAC within the extracellular domain of α-ENaC, as obtained using Discovery Studio. The interaction map depicts hydrogen bonds, π-related interactions, and van der Waals contacts between ICAC and surrounding amino acid residues, highlighting a putative ligand–protein interaction interface. Colors and dashed lines indicate interaction types: pale green, van der Waals; green, conventional hydrogen bonds; light green, carbon hydrogen bonds; orange, π–anion interactions; pink, π–π T-shaped interactions; and light pink, π–alkyl interactions.

Building on this hypothesis, molecular docking analysis was performed to further explore the potential binding interactions between ICAC and α-ENaC. Docking simulations were conducted using Discovery Studio, and the predicted binding energies were used as an indicator of the relative strength of ligand–protein interactions. In general, negative binding energy values suggest thermodynamically favorable binding, with docking scores below −5.0 kcal/mol commonly interpreted as moderate affinity and values below −7.0 kcal/mol indicative of stronger and more stable interactions [26]. As shown in Figure 6, the two-dimensional interaction map depicts a plausible binding mode of ICAC within the extracellular domain of α-ENaC. The molecular docking results demonstrated that ICAC exhibits favorable binding affinity toward α-ENaC, with a calculated binding energy of approximately −8.1 kcal/mol, suggesting a stable interaction within the extracellular domain. ICAC is predicted to occupy a shallow surface pocket and to be stabilized by a combination of hydrogen bonds, π-related interactions, and van der Waals contacts. Specifically, conventional hydrogen bonds were identified with GLN356, ASP310, PHE435, and TYR444, contributing to ligand stabilization. In addition, ASP310 and ASP363 participate in π–anion interactions, providing further electrostatic stabilization of the ligand–protein complex. A π–π T-shaped interaction with TYR436 and a π–alkyl interaction with PRO408 were also observed, further supporting ligand binding. Moreover, residues including ILE434, PRO359, GLU358, HIS354, ASN312, LYS311, ALA360, PHE361, HIS418, and ASP364, among others, form a network of van der Waals interactions, creating a hydrophobic environment that facilitates ligand accommodation within the binding pocket.

## 3. Discussion

Ang II plays a critical role in maintaining physiological functions, including sodium and water homeostasis and vascular tone; however, its dysregulation contributes to the development of pathological conditions such as atherosclerosis and hypertension. ROS are key mediators of Ang II-induced renal remodeling, as their excessive production triggers oxidative stress (Figure 1e), which in turn promotes inflammation and tissue damage. Mitochondrial depolarization induced by Ang II contributes to oxidative stress, which activates signaling pathways that enhance the production of cytokines such as IL-1β, IL-6, and TNF-α. These inflammatory mediators further exacerbate cellular damage, leading to hypertension and organ remodeling. By reducing ROS and mitochondrial depolarization, AAE treatment may help regulate cytokine release, thereby mitigating inflammation and its associated pathological consequences (Figure 2c–e). Consistent with these findings, our results demonstrated that ICAC, the main active component of AAE, also possesses the ability to suppress ROS production and alleviate inflammation. Previous studies have shown that isochlorogenic acid A (ICAA) reduces liver fibrosis by modulating the TLR4/NF-κB signaling axis [27], while chlorogenic acid mitigates acute kidney injury by blocking NLRP3 inflammasome-mediated pyroptosis [28]. Additionally, ICAC suppresses inflammatory cell hyperactivation and inflammation-induced synovial proliferation through the Erk/JNK/NF-κB pathway [29]. Collectively, these findings highlight the therapeutic potential of these bioactive compounds in modulating the inflammatory and pyroptosis pathways. According to our results, AAE and ICAC inhibited the NLRP3 inflammasome, thereby attenuating the pyroptosis pathway (Figure 3). The enhanced effect of AAE, compared with ICAC, may be attributed to its composition as a complex mixture of bioactive compounds, which collectively exert broader regulatory effects on Ang II–induced pyroptosis.

Ion channels are integral membrane proteins that regulate the influx and efflux of ions, thereby controlling membrane potential and cellular volume. In addition, chlorogenic acid has been reported to influence potassium efflux, suggesting that its antifungal activity may be associated with interactions with ion channels [30]. Following Ang II stimulation, cells experience not only membrane depolarization but also excessive sodium efflux into the extracellular space and increased potassium influx, which further disrupts ionic homeostasis. However, growing evidence supports a direct role of Ang II in modulating epithelial cell function [31]. The epithelial sodium channel (ENaC) is composed of three homologous subunits (α, β, and γ), and the coexpression of all three is required for maximal channel activity. Among these, the α-subunit plays a pivotal role in forming a functional ion channel [32]. Regarding the direct actions of Ang II on ENaC, accumulating evidence indicates that Ang II enhances distal sodium transport by upregulating α-ENaC expression and by increasing channel activity through modulation of its open probability [33,34,35]. More recently, Ang II has also been shown to directly facilitate the translocation of α-ENaC to the apical plasma membrane, thereby increasing the number of functionally active channels in vivo [36].

Chronic Ang II infusion markedly enhanced both the expression and activity of ENaC, the principal sodium transporter in the distal tubules. ENaC, located on the apical membrane, mediates the entry of Na^+^ from the tubular lumen into epithelial cells. Subsequently, Na^+^ is actively transported across the basolateral membrane, which interfaces with the interstitium and bloodstream, by the Na^+^/K^+^-ATPase in exchange for potassium (K^+^). Previous studies have demonstrated that gene-targeted disruption of angiotensin II type 1 receptor-associated protein exacerbates Ang II-induced hypertension through pathological activation of renal tubular AT1R [37]. Notably, circulating and urinary aldosterone levels remained comparable, and neither blood pressure responses to aldosterone nor renal ENaC expression were affected. These findings suggest that Ang II directly stimulates ENaC in the distal tubules, thereby promoting sodium retention through an aldosterone-independent mechanism [38]. In Figure 4a, AAE and ICAC were found to modulate Na^+^/K^+^-ATPase activity and counteract the effects of Ang II, which impairs α-ENaC ubiquitination through the inhibition of Nedd4-2. This results in sustained α-ENaC expression on the apical membrane, promoting increased sodium reabsorption. Ang II disrupts the balance of α-ENaC ubiquitination by suppressing Nedd4-2 activity, resulting in prolonged α-ENaC retention at the apical membrane and consequently promoting increased sodium reabsorption. Following Ang II stimulation, SGK1 inhibits Nedd4-2–mediated ubiquitination of α-ENaC, leading to its stabilization at the apical membrane and sustained sodium influx. Figure 4b showed that Ang II markedly activated SGK1, thereby suppressing Nedd4-2 activity. However, this effect was reversed by AAE and its active component, ICAC, highlighting their potential role in restoring proper α-ENaC regulation (Figure 5a). Treatment with high doses of AAE suppresses this regulatory mechanism, helping to restore ion channel homeostasis and exerting a protective effect on renal epithelial cells (Figure 5b).

A total of 86 traditional Chinese medicinal herbs have been statistically identified as containing isochlorogenic acids, with the Asteraceae family being recognized as a vibrant source [39]. Consistent with previous studies, isochlorogenic acids exhibit stronger cytochrome c–reducing capacity than chlorogenic acid and demonstrate concentration-dependent regulation of mitochondrial function in renal systems [40]. In the study, AAE and its principal active compound, ICAC, significantly modulated mitochondrial potential, exhibited anti-inflammatory activity, and contributed to the inhibition of pyroptosis (Figure 2a,b). Notably, the predicted binding site is located outside the canonical ion conduction pathway, implying that ICAC may not directly block sodium permeation. Instead, this interaction pattern raises the possibility that ICAC could influence α-ENaC function indirectly; for example, by affecting channel conformation or regulatory accessibility. Although these docking results are predictive in nature, they are consistent with the experimental observations showing altered α-ENaC expression or activity following ICAC treatment, and they provide a structural basis for the proposed modulatory effects of ICAC on ENaC (Figure 6). Based on our findings, AAE and its active component, ICAC, appear to play a significant role in regulating ion channel activity. AAE inhibition reduces α-ENaC activity in AngII-induced conditions not only by lowering the number of functional channels on the membrane but also by decreasing α-ENaC open probability. This underscores the need for further investigation into the role of noncanonical aldosterone signaling pathways in regulating renal sodium handling and blood pressure.

## 4. Materials and Methods

### 4.1. Preparation of Artemisia indica Willd. Aqueous Extract (AAE) and Functional Components Assay

*Artemisia indica* Willd. was obtained in Taichung, Taiwan, and authenticated by the Herbarium of the Taiwan Biodiversity Research Institute (No. 051489). Dried leaves of *Artemisia indica* Willd. (300 g) were extracted with hot water (100 °C, 1500 mL) for 1 h. The resulting aqueous extract was subsequently concentrated under reduced pressure and freeze-dried. The final yield of *Artemisia indica* Willd. aqueous extract (AAE) was approximately 12.2% of the dry plant material. The lyophilized extract was stored at −80 °C until further experimental use.

The functional constituents of AAE were quantified using established spectrophotometric assays. Total polyphenol content was determined using the Folin–Ciocalteu method with gallic acid as the calibration standard. AAE (0.1 mg) was dissolved in 1 mL of distilled water, followed by the addition of Folin–Ciocalteu reagent (2 N, 0.5 mL). After 3 min, 3 mL of 2% Na_2_CO_3_ was added, and the mixture was incubated for 15 min with intermittent mixing. Absorbance was then measured at 750 nm using a spectrophotometer. Total flavonoid content was measured according to the Jia method using rutin as the reference compound. AAE solution (0.5 mL, 1 mg/mL) was mixed with 1.25 mL of distilled water and 75 μL of 5% NaNO_2_. After 6 min, 150 μL of 10% AlCl_3_·6H_2_O was added, and the mixture was allowed to react for 5 min. Subsequently, 0.5 mL of 1 M NaOH and 2.5 mL of distilled water were added, and absorbance was recorded immediately at 510 nm. Total anthocyanin content was determined using the Fuleki and Francis pH differential method. AAE solution (1 mg/mL) was diluted with pH 1.0 and pH 4.5 buffer solutions, and absorbance was measured at 535 nm using distilled water as the blank. The anthocyanin concentration was calculated from the difference in absorbance between the two buffer conditions. The quantified functional components of AAE are summarized in Table 1.

### 4.2. HPLC-ESI-MS/MS Profiling and Identification of Phytochemical Constituents in AAE

The HPLC/electrospray ionization (ESI) mass spectrometric analysis was performed using an Agilent 1260 Infinity HPLC system coupled with a 6420 triple quadrupole mass spectrometer (Agilent Technologies, Santa Clara, CA, USA) operating in negative ionization mode, as described in a previous report with minor modifications [41]. The AAE sample was filtered through a 0.45 μm membrane filter prior to injection into a Symmetry C18 column (2.1mm × 150 mm, 3.5 μm; Waters Corporation, Milford, MA, USA), which was connected to a guard column (SecurityGuard C18 (ODS), 4 mm × 3.0 mm ID; Phenomenex Inc., Torrance, CA, USA) and maintained at 35 °C. The mobile phase consisted of solvent A (water containing 0.1% formic acid) and solvent B (acetonitrile containing 0.1% formic acid), with a flow rate of 0.3 mL/min. A linear gradient elution was applied as follows: 10–15% B (0–1 min), 15–30% B (1–3 min), 30–50% B (3–23 min), 50–95% B (23–43 min), followed by 95% B isocratic elution for 10 min. UV absorption spectra were recorded over a range of 210–600 nm using an in-line photodiode array (PDA) detector, with monitoring wavelengths set at 254, 270, and 320 nm. Mass spectrometric detection was conducted using a triple quadrupole (QQQ) mass spectrometer under the following conditions: nitrogen was used as both the drying gas (9 L/min) and nebulizing gas (35 psi), with a drying gas temperature of 325 °C. The capillary voltage was set at 3500 V, the fragmentor voltage at 100 V, and the collision energy at 25 V. MS/MS data were acquired in product ion scan mode, where quadrupole 1 (Q1) selected precursor ions and quadrupole 2 (Q2) generated fragment ions via collision-induced dissociation (CID). Product ions were scanned over an *m*/*z* range of 100–1000 with a scan time of 200 ms per cycle. Compound identification was performed based on retention times, UV–Vis absorption maxima, deprotonated molecular ions ([M − H]^−^), and MS/MS fragmentation patterns, with the major fragment ions summarized in Table S1. The identified compounds and their retention times were 3-caffeoylquinic acid (2.99 min), 4-caffeoylquinic acid (4.53 min), 5-caffeoylquinic acid (4.87 min), caffeic acid (5.32 min), apigenin 6,8-C-pentoside-hexoside (5.81 min), apigenin 6,8-di-C-pentoside (6.39 min), rutin (6.67 min), quercetin-3-O-glucoside (7.12 min), isochlorogenic acid B (ICAB) (8.16 min), ICAA (8.44 min), and ICAC (9.37 min) (Figure S1 and Table S1). MS/MS fragmentation patterns were used as the primary basis for distinguishing positional isomers, and established diagnostic features, including characteristic fragment ions, were interpreted according to well-recognized fragmentation rules for caffeoylquinic acid derivatives [42]. In our study, compound identification was supported by comparison of retention times and fragmentation behavior with previously reported data for compounds within the *Artemisia* genus [41]. The three isochlorogenic acid isomers (Peaks 9–11) all exhibited identical deprotonated molecular ions ([M − H]^−^) at *m*/*z* 515; however, their identities as ICAB, ICAA, and ICAC were assigned based on their characteristic product ions at *m*/*z* 191, 179, 173, and 135, consistent with previously reported fragmentation patterns [41,42]. For the semi-quantitative estimation of the identified constituents, peak area normalization was employed. The relative abundance of each compound was calculated as a percentage (%) of the total chromatographic peak area monitored by the DAD detector. As external calibration using authentic standards was not performed, the reported values represent relative proportions within the extract rather than absolute concentrations. As shown in Table 1, the predominant constituents were ICAC (21.26 ± 1.2%), followed by ICAB (17.93 ± 0.8%) and ICAA (13.74 ± 0.5%).

### 4.3. Cell Culture and Viability Assessment

Rat renal proximal tubular epithelial cells (NRK-52E) were obtained from the Bioresource Collection and Research Center (BCRC, Taiwan). Cells were cultured in Dulbecco’s Modified Eagle Medium (DMEM) supplemented with 5% fetal bovine serum and 1% penicillin–streptomycin, and maintained at 37 °C in a humidified atmosphere containing 5% CO_2_. To evaluate cytotoxicity, NRK-52E cells were treated with various concentrations of AAE (0, 5, 10, 20, 50, and 100 μg/mL), Ang II (0, 1, 2, and 10 μM) or ICAC (0, 1, 5, 10, 20 and 50 μΜ) for 24 h. The concentration of ICAC used for comparison was estimated based on its relative abundance in AAE. ICAC constituted 21.26% of AAE. Accordingly, at an AAE concentration of 20 µg/mL, the corresponding ICAC concentration was calculated to be 4.252 µg/mL, which is equivalent to approximately 8.24 µM based on its molecular weight. For combination treatments, cells were pretreated with AAE (10 or 20 μg/mL) or ICAC (8.24 µM) for 1 h, followed by exposure to Ang II (1 μM) for an additional 23 h. After treatment, cell viability was determined by staining with propidium iodide (PI, 10 μg/mL; Sigma-Aldrich, St. Louis, MO, USA) and analyzed using the Muse™ Cell Analyzer (EMD Millipore, Merck Life Sciences, Darmstadt, Germany). In the scatter plots, the *x*-axis represents PI fluorescence intensity and the *y*-axis represents cell size. Gated regions were used to quantify the proportion of viable cells. The percentage of live cells was further quantified and presented as bar graphs with statistical analysis. All treatments were performed in at least three independent biological replicates.

### 4.4. Reactive Oxygen Species (ROS) Content Analysis

To determine intracellular ROS levels, NRK-52E cells were seeded in 6-well plates and treated as described above. After 24 h of total incubation, intracellular ROS were measured using the Muse Oxidative Stress Kit (Luminex, Austin, TX, USA) according to the manufacturer’s protocol. Quantification was performed using the Muse™ Cell Analyzer [43]. 

### 4.5. Determination of Mitochondrial Membrane Potential

NRK52E cells were seeded into 6-well cell culture plates and treated as described above. After harvesting, cells were detached using trypsin-EDTA and analyzed for mitochondrial membrane potential (Δψm) using the Muse™ MitoPotential Kit (Muse, MCH100110) according to the manufacturer’s instructions. 7-AAD is a membrane-impermeant DNA-binding dye that serves as an indicator of cell membrane integrity and cell death. It is excluded from viable and early apoptotic cells but penetrates late apoptotic and dead cells, thereby enabling discrimination of non-viable populations. In parallel, the MitoPotential dye accumulates within the inner mitochondrial membrane in a membrane potential-dependent manner. Cells with intact mitochondrial membrane potential exhibit strong fluorescence, whereas depolarized mitochondria show reduced dye accumulation. Δψm was assessed by flow cytometry [44].

### 4.6. IL-6, IL-18, or IL-1β Cytokines Assay

Following treatment, the cell culture supernatants were collected to assess the levels of cytokines. The concentrations of IL-6, IL-18, and IL-1β were measured using enzyme-linked immunosorbent assay (ELISA) kits (ELISA MAX™ Deluxe Sets, BioLegend, San Diego, CA, USA) according to the manufacturer’s instructions [45].

### 4.7. Western Blotting

After treatment, the cells were washed with phosphate-buffered saline and then lysed in a buffer (Cell Signaling Technology, Danvers, MA, USA) containing a protease inhibitor cocktail. Cell lysates were sonicated and centrifuged to remove debris. Protein concentrations were determined using the Dual-Range™ BCA Protein Assay Kit (Energenesis Biomedical Co., Ltd., Taipei, Taiwan). Equal amounts of protein were resolved by 10% or 12% SDS-PAGE and transferred onto 0.45 μm PVDF membranes (Immobilon-P, Merck Millipore, Burlington, MA, USA). Membranes were blocked with 5% non-fat dry milk in TBS-T (Tris-buffered saline with 0.1% Tween-20) for 1 h at 4 °C and then incubated overnight at 4 °C with the following primary antibodies: AT1R (sc-515884), p-PKCα (sc-517540), PKCα (sc-8393), NF-κB (sc-8008), ASC (sc-514414), Caspase-1 (sc-56036), GSDMD (sc-81868), IL-1β (sc-12742) SGK1(sc-28338), NEDD4-2 (sc-514954), and α-ENaC (sc-22239) were purchased from Santa Cruz Biotechnology (Santa Cruz, CA, USA), NLRP3 (A12694) were purchased from Abclonal (Woburn, MA, USA), and anti-β-actin (A5441) from Sigma–Aldrich (St. Louis, MO, USA). After three washes with TBS-T, membranes were incubated with appropriate secondary antibodies for 1 h at room temperature. Protein bands were visualized using enhanced chemiluminescence reagents (Millipore, Burlington, MA, USA) and captured using a LAS-4000 Luminescent Image Analyzer (Fujifilm Corporation, Tokyo, Japan).

### 4.8. Na/K-ATPase Activity

Na^+^/K^+^-ATPase activity was measured using the Na^+^/K^+^-ATPase Activity Assay Kit (Cohesion Biosciences, Suzhou, China, Catalog #CAK1019) according to the manufacturer’s instructions. Briefly, NRK52E cells were lysed in assay buffer, followed by sonication and centrifugation at 8000× *g* for 10 min at 4 °C. The amount of inorganic phosphate released was determined colorimetrically and expressed as μmol per mg of protein.

### 4.9. Immunocytochemistry Staining

NRK-52E cells were fixed with 4% paraformaldehyde, permeabilized using Triton X-100 for 20 min, and subsequently blocked with blocking buffer for 1 h at room temperature. Cells were incubated overnight at 4 °C with the primary antibody against α-ENaC (sc-22239) on a shaker. After three washes with phosphate-buffered saline, cells were incubated with fluorescently labeled secondary antibodies for 2 h at room temperature in the dark. Nuclei were counterstained with 4′,6-diamidino-2-phenylindole (DAPI) to visualize cellular architecture [46].

### 4.10. Immunoprecipitation

Following treatment, NRK-52E cells were lysed, and magnetic beads (Bio-Rad Laboratories, Inc., Hercules, CA, USA) were incubated with the appropriate primary antibody (α-ENaC) for 10 min at room temperature to form the bead–antibody complex. After incubation with samples, the beads were washed four times to remove nonspecific binding. The bound proteins were eluted by boiling in loading buffer for 5 min and subsequently analyzed by Western blotting.

### 4.11. Molecular Docking Analysis

Molecular docking analysis was performed to investigate the potential binding interactions between ICAC and α-ENaC. The three-dimensional structure of human ENaC was retrieved from the Protein Data Bank (PDB ID: 6WTH; https://www.rcsb.org/). Specifically, Chain A, annotated as the human amiloride-sensitive sodium channel subunit alpha (SCNN1A), was selected for analysis. Prior to docking, heteroatoms, water molecules, and redundant subunits were removed to prepare the receptor model. The chemical structure of ICAC was obtained from the PubChem database at the National Center for Biotechnology Information (https://pubchem.ncbi.nlm.nih.gov/) and converted into PDB format for docking analysis. Molecular docking was conducted using the SwissDock server (https://www.swissdock.ch/) by submitting the prepared α-ENaC protein and ICAC ligand structures. Docking results were generated as compressed files containing multiple predicted binding poses, which were subsequently extracted and analyzed. The docked complexes were visualized and further analyzed using Discovery Studio (Dassault Systèmes BIOVIA, San Diego, CA, USA) software to identify hydrogen bonds and non-bonded interactions between ICAC and amino acid residues within the predicted binding region, as well as to generate high-quality molecular interaction images [47,48].

### 4.12. Statistical Analysis

Data are expressed as mean ± standard deviation (SD). Statistical analyses were performed using SigmaPlot 10.0 (Systat Software, Inc., San Jose, CA, USA). Comparisons between two groups were conducted using Student’s *t*-test. For multiple group comparisons, one-way analysis of variance (ANOVA) followed by Tukey’s post hoc test was applied. A *p*-value < 0.05 was considered statistically significant.

## 5. Conclusions

**Figure 7:**
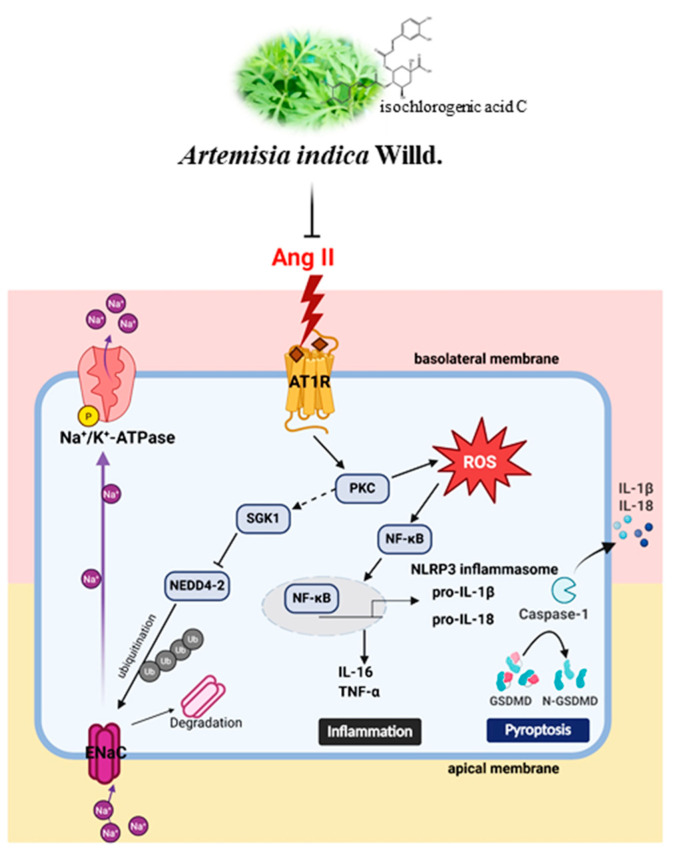
Proposed mechanism by which *Artemisia indica* Willd. aqueous extract (AAE) and isochlorogenic acid C (ICAC) attenuate Ang II-induced renal epithelial cell injury. Angiotensin II (Ang II) activates the angiotensin II type 1 receptor (AT1R), leading to protein kinase C (PKC) activation and increased reactive oxygen species (ROS) production, which subsequently activates nuclear factor kappa B (NF-κB) and promotes the release of pro-inflammatory cytokines, including interleukin-6 (IL-6) and tumor necrosis factor-α (TNF-α). In parallel, Ang II induces NLRP3 inflammasome activation, resulting in caspase-1 activation, gasdermin D (GSDMD) cleavage, and the release of interleukin-1β (IL-1β) and interleukin-18 (IL-18), leading to pyroptosis. Ang II also disrupts sodium homeostasis by enhancing epithelial sodium channel (ENaC) activity and Na^+^/K^+^-ATPase function. Serum/glucocorticoid-regulated kinase 1 (SGK1) inhibits neural precursor downregulated protein 4-2 (Nedd4-2), thereby reducing ENaC ubiquitination and promoting sodium reabsorption. AAE and ICAC attenuate these pathological processes by suppressing AT1R/PKC/NF-κB signaling, reducing ROS production, inhibiting NLRP3 inflammasome activation and pyroptosis, and restoring sodium homeostasis through modulation of the SGK1/Nedd4-2/ENaC axis.

This study demonstrates that AAE and its major constituent, ICAC, exert multifaceted protective effects against Ang II-induced renal injury. AAE and ICAC attenuated oxidative stress, mitochondrial dysfunction, and proinflammatory cytokine release, while suppressing AT1R/PKC/NF-κB activation and NLRP3 inflammasome-mediated pyroptosis. Furthermore, they restored sodium homeostasis by modulating the SGK1/Nedd4-2/ENaC axis, thereby limiting Ang II-driven sodium retention. Molecular docking analysis indicated that ICAC interacts with the extracellular domain of α-ENaC via multiple non-covalent interactions, which may contribute to its potential modulatory effects. In summary, these findings highlight the therapeutic potential of AAE and ICAC as natural phytochemicals for managing hypertension-related renal injury through the integrated regulation of oxidative, inflammatory, and ion transport pathways (Figure 7).

## Figures and Tables

**Table 1:** Identification and semi-quantitative composition of phytochemical compounds in AAE determined by HPLC–ESI–MS/MS.

Polyphenolic Compound	Peak No. ^a^	AAE (%)	Reference
ICAB	9	17.93 ± 0.8	[41]
ICAA	10	13.74 ± 0.5	
ICAC	11	21.26 ± 1.2	
Total Polyphenol (Folin–Ciocalteu Method)		78.9 ± 13.1	
Total Flavonoid (Jia Method)		18.9 ± 1.5	
Total Anthocyanin (Fuleki and Francis Method)		5.2 ± 2.2	

^a^ Phenolic compounds correspond to peaks as in HPLC-ESI-MS/MS chromatogram of eleven kinds of polyphenols.

## Data Availability

Data is contained within the article.

## Supplementary Materials

The following supporting information can be downloaded at: https://www.mdpi.com/article/10.3390/plants15091405/s1, Figure S1: Identification and quantification of phytochemical constituents in *Artemisia indica* Willd. aqueous extract (AAE) by HPLC-ESI-MS/MS. Table S1: Identification and semi-quantitative composition of polyphenolic compounds in *Artemisia indica* Willd. aqueous extract (AAE) determined by HPLC–ESI–MS/MS.

## Abbreviations

Ang II	Angiotensin II
AT1R	Angiotensin II type 1 receptor
ACE	Angiotensin-converting enzyme
AAE	*Artemisia indica* Willd. aqueous extract
CKD	Chronic kidney disease
ENaC	Epithelial sodium channel
IL-1β	Interleukin-1 beta
IL-6	Interleukin-6
ICAC	Isochlorogenic acid C
ICAA	Isochlorogenic acid A
ICAB	Isochlorogenic acid B
Nedd4-2	Neural precursor cell-expressed developmentally downregulated gene 4-like
PKC	Protein kinase C
ROS	Reactive oxygen species
RAS	Renin–angiotensin system
