# Supplementary material for: Artemisia indica Willd. Extract Regulate NLRP3 Inflammasome and ENaC Trafficking in Angiotensin II-Stimulated Renal Tubular Cells
Source: Plants (Basel). 2026 May 4;15(9):1405. doi: 10.3390/plants15091405 (PMC13164963; doi:10.3390/plants15091405)
Supplement: Supplementary file 1 [file plants-15-01405-s001.zip › plants-4264120-supplementary.pdf]

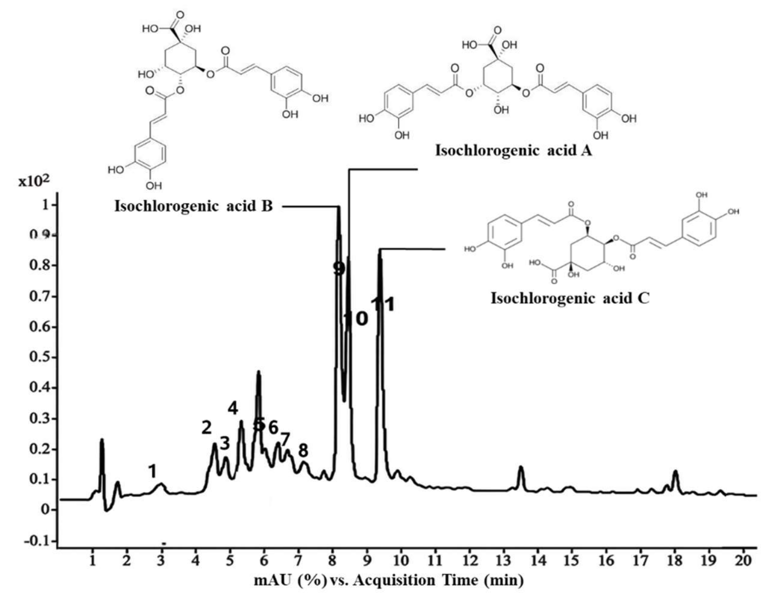

Figure S1. Identification and quantification of phytochemical constituents in *Artemisia indica* Willd. aqueous extract (AAE) by HPLC-ESI-MS/MS. Peaks 1-11 represent the identified phenolic compounds, including caffeoylquinic acids, isochlorogenic acids, and flavonoid glycosides (Table S1 for details). Chemical structures of major isochlorogenic acid isomers (isochlorogenic acids A-C) are illustrated.

Table S1. Identification and semi-quantitative composition of polyphenolic compounds in *Artemisia indica* Willd. aqueous extract (AAE) determined by HPLC–ESI–MS/MS.

**Polyphenolic compound**[a]	**Peak No.**[b]	**UV-Vis λmax (nm)**	**MS [M−H]⁻ (m/z)**	**MS/MS fragment ions (m/z)**	**AAE (%)**[c]	**Reference**
Caffeoylquinic acid derivatives						[42]
3-Caffeoylquinic acid	1	324, 294 sh	353	191, 179, 135	2.22 ± 0.5	
4-Caffeoylquinic acid	2	324, 292 sh	353	191	5.02 ± 0.1	
5-Caffeoylquinic acid	3	324, 296 sh	353	191, 173, 179, 135	1.88 ± 0.7	
Caffeic acid	4	322, 294 sh	179	135	5.58 ± 1.2	
Flavonoid glycosides						[41]
Apigenin 6,8-C-pentoside-hexoside	5	328, 270	563	353, 383, 473, 443	8.05 ± 0.2	
Apigenin 6,8-di-C-pentoside	6	326	533	353	1.97 ± 0.3	
Rutin	7	254, 352	609	300	1.98 ± 1.2	
Quercetin-3-O-glucoside	8	330	463	300, 271	1.97 ± 0.5	
isochlorogenic acid derivatives						[41]
isochlorogenic acid B	9	322, 296 sh, 242	515	179, 173, 191, 135	17.93 ± 0.8	
isochlorogenic acid A	10	326, 298 sh, 240	515	191, 179, 135, 173	13.74 ± 0.5	
isochlorogenic acid C	11	326, 298 sh, 242	515	173, 179, 191, 135	21.26 ± 1.2	

[a]Compound identification was based on retention time, UV–Vis absorption maxima (λ_max), deprotonated molecular ions ([M−H]⁻), and characteristic MS/MS fragmentation patterns. [b]Peak numbers correspond to those shown in Figure S1. [c]Relative contents were calculated by peak area normalization from DAD chromatograms and are expressed as percentage (%) of the total chromatographic peak area. Data are presented as mean ± SD (n ≥ 3). These values represent relative abundances rather than absolute concentrations.

References

41. Han, B.; Xin, Z.; Ma, S.; Liu, W.; Zhang, B.; Ran, L.; Yi, L.; Ren, D. Comprehensive characterization and identification of antioxidants in Folium Artemisiae Argyi using high-resolution tandem mass spectrometry. *J. Chromatogr. B* **2017**, *1063*, 84–92.
42. Clifford, M.N.; Johnston, K.L.; Knight, S.; Kuhnert, N. Hierarchical Scheme for LC-MSn Identification of Chlorogenic Acids. *J. Agric. Food Chem.* **2003**, *51*, 2900–2911.
